# Loss of arginine vasopressin- and vasoactive intestinal polypeptide-containing neurons and glial cells in the suprachiasmatic nucleus of individuals with type 2 diabetes

**DOI:** 10.1007/s00125-019-4953-7

**Published:** 2019-07-20

**Authors:** Rick Hogenboom, Martin J. Kalsbeek, Nikita L. Korpel, Paul de Goede, Marit Koenen, Ruud M. Buijs, Johannes A. Romijn, Dick F. Swaab, Andries Kalsbeek, Chun-Xia Yi

**Affiliations:** 1 Department of Endocrinology and Metabolism, Amsterdam University Medical Centers (UMC), Location AMC, Meibergdreef 9, 1105 AZ Amsterdam, the Netherlands; 2 Laboratory of Endocrinology, Amsterdam University Medical Centers (UMC), University of Amsterdam, Amsterdam Gastroenterology and Metabolism, Amsterdam, the Netherlands; 3 Netherlands Institute for Neuroscience, an Institute of the Royal Netherlands Academy of Arts and Sciences, Amsterdam, the Netherlands; 4 Department of Cell Biology and Physiology, Institute for Biomedical Research, Universidad Nacional Autonoma de Mexico, Mexico City, Mexico; 5 Department of Medicine, Amsterdam University Medical Centers (UMC), University of Amsterdam, Amsterdam, the Netherlands

**Keywords:** Astroglial cells, Biological clock, Insulin resistance, Microglia, Neurotensin, Rhythmicity, Type 2 diabetes mellitus

## Abstract

**Aims/hypothesis:**

The central pacemaker of the mammalian biological timing system is located within the suprachiasmatic nucleus (SCN) in the anterior hypothalamus. Together with the peripheral clocks, this central brain clock ensures a timely, up-to-date and proper behaviour for an individual throughout the day–night cycle. A mismatch between the central and peripheral clocks results in a disturbance of daily rhythms in physiology and behaviour. It is known that the number of rhythmically expressed genes is reduced in peripheral tissue of individuals with type 2 diabetes mellitus. However, it is not known whether the central SCN clock is also affected in the pathogenesis of type 2 diabetes. In the current study, we compared the profiles of the SCN neurons and glial cells between type 2 diabetic and control individuals.

**Methods:**

We collected post-mortem hypothalamic tissues from 28 type 2 diabetic individuals and 12 non-diabetic control individuals. We performed immunohistochemical analysis for three SCN neuropeptides, arginine vasopressin (AVP), vasoactive intestinal polypeptide (VIP) and neurotensin (NT), and for two proteins expressed in glial cells, ionised calcium-binding adapter molecule 1 (IBA1, a marker of microglia) and glial fibrillary acidic protein (GFAP, a marker of astroglial cells).

**Results:**

The numbers of AVP immunoreactive (AVP-ir) and VIP-ir neurons and GFAP-ir astroglial cells in the SCN of type 2 diabetic individuals were significantly decreased compared with the numbers in the SCN of the control individuals. In addition, the relative intensity of AVP immunoreactivity was reduced in the individuals with type 2 diabetes. The number of NT-ir neurons and IBA1-ir microglial cells in the SCN was similar in the two groups.

**Conclusions/interpretation:**

Our data show that type 2 diabetes differentially affects the numbers of AVP- and VIP-expressing neurons and GFAP-ir astroglial cells in the SCN, each of which could affect the daily rhythmicity of the SCN biological clock machinery. Therefore, for effectively treating type 2 diabetes, lifestyle changes and/or medication to normalise central biological clock functioning might be helpful.

**Electronic supplementary material:**

The online version of this article (10.1007/s00125-019-4953-7) contains peer-reviewed but unedited supplementary material, which is available to authorised users.

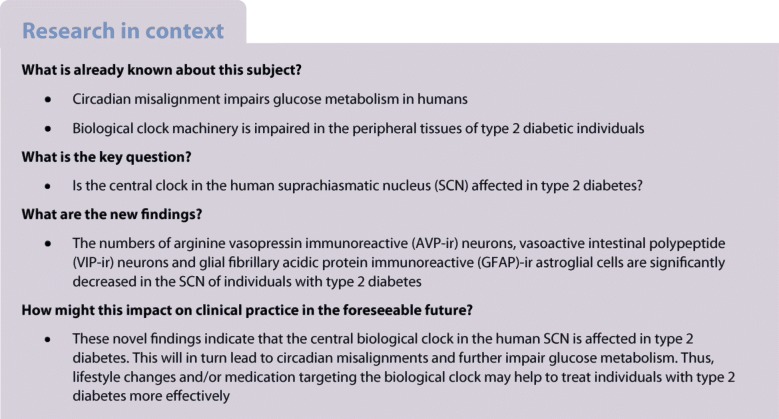



## Introduction

In mammals, the circadian timing system plays a critical role in coordinating the daily and seasonal rhythmicity of all physiological and behavioural processes in the body. The master pacemaker of this timing system is located in the suprachiasmatic nucleus (SCN) in the hypothalamus. Multiple types of neurons are involved in the SCN neuronal network [1]. In rodents, these mainly include the vasoactive intestinal polypeptide (VIP)-producing and the arginine vasopressin (AVP)-producing neurons [1], and in addition to these, humans also possess neurotensin (NT)-containing neurons [2].

Type 2 diabetes mellitus is characterised by hyperglycaemia and insulin resistance. Glucose homeostasis and insulin sensitivity are tightly controlled by the circadian timing system, mainly through balancing sympathetic and parasympathetic outputs from the hypothalamus [3]. Previous studies have shown that impaired insulin secretion in prediabetic animal models results in decreased insulin signalling in the hypothalamus, leading to decreased inhibition of glucose production in the liver and impaired glucose uptake [4, 5]. Evidence is accumulating for a link between circadian misalignment, for example, by sleep deprivation, and profound disruptions in blood glucose and insulin levels [6]. Thus far, few studies have investigated peripheral clock machinery in individuals with type 2 diabetes [7], and it has never been studied whether the central clock in the SCN itself is affected by type 2 diabetes. The current study aimed to profile and compare SCN neurons, especially the ones producing AVP, VIP and NT, as well as the astroglial cells (using glial fibrillary acidic protein, GFAP, as a marker) and microglia (using ionised calcium-binding adapter molecule 1, IBA1, as a marker) in control and type 2 diabetic individuals.

## Methods

### Donors

Post-mortem hypothalamic tissues from 28 type 2 diabetic and 12 non-diabetic control individuals were obtained from the Netherlands Brain Bank, through autopsy approved by the Medical Ethic Committee of the VU Medical Center, the Netherlands. Individuals with Braak stage V–VI or clinically diagnosed severe dementia were excluded [8]. Sex, age, time/month of death were similar between groups (Electronic supplementary material [ESM] Table 1). Data on the latest post-absorptive blood glucose and HbA_1c_, although not complete, as indications of glycaemic control are presented in ESM Table 1. Other donor details, including post-mortem delay, clinical diagnosis, diagnosed high blood pressure, insulin treatment and cause of death are provided in ESM Table 1.

### Immunohistochemistry and image analysis

Immunohistochemistry for AVP-ir, VIP-ir, NT-ir, GFAP-ir and IBA1-ir cells in the SCN was performed (see ESM Methods). Images were analysed by the Fiji image processing program, an ImageJ distribution (Madison, WI, USA). The soma number and relative intensity of immunoreactivity for AVP-ir, VIP-ir and NT-ir neurons; the number of GFAP-ir astroglial cells and the soma number/soma size for IBA1-ir microglia (per section) were quantified by a blinded investigator (R. Hogenboom) (see ESM Methods for further details).

The numbers of AVP-ir and VIP-ir cells at each level of the SCN were plotted along the rostral–caudal axis for all control individuals. To profile the other cells, for each individual we selected consecutive sections next to the one that contained the highest number of AVP-ir cells (ESM Fig. 1).

### Statistics

All data are presented as means ± SEM. Comparisons between control and type 2 diabetic individuals were analysed by Student’s *t* test. A *p* value of <0.05 was considered to be significant. Daily rhythmicity and monthly variation in the number of AVP-ir, VIP-ir, NT-ir, GFAP-ir and IBA1-ir cells in the SCN was assessed using cosinor analysis SigmaPlot 14.0 software (SPSS, Chicago, IL, USA) (see ESM Methods for further details).

## Results

**Fig. 1:**
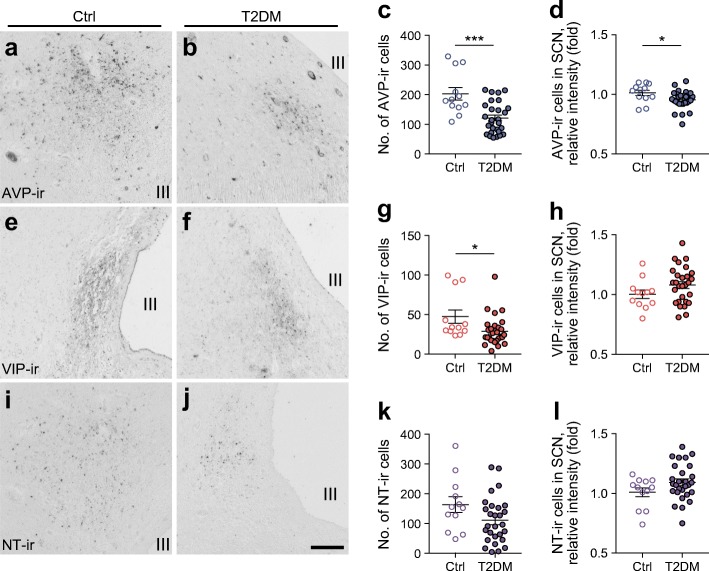
AVP-ir neurons, VIP-ir neurons and NT-ir neurons in the SCN of non-diabetic and type 2 diabetic individuals. Representative images of AVP-ir (**a**, **b**), VIP-ir (**e**, **f**) and NT-ir (**i**, **j**) neurons in the SCN of non-diabetic (Ctrl) and type 2 diabetic (T2DM) individuals. Comparison of the number of soma of the AVP-ir (**c**), VIP-ir (**g**) and NT-ir (**k**) neurons, the relative intensity of immunoreactivity of the AVP-ir (**d**), VIP-ir (**h**) and NT-ir (**l**) neurons (shown as fold of Ctrl) in the SCN of non-diabetic and type 2 diabetic individuals. Data are presented as mean ± SEM. *p<0.05, ***p<0.001. III, third cerebral ventricle. Scale bar, 300 μm

AVP-ir neurons were mainly distributed in the dorsal SCN, while VIP-ir neurons were mainly found in the ventral and central SCN (Fig. 1, ESM Fig. 1). NT-ir neurons were visible in the dorsomedial and ventral SCN (Fig. 1). When considering all donors (*n* = 40), no correlations were found between the numbers of SCN AVP-ir, VIP-ir and NT-ir neurons and age, post-mortem delay, post-absorptive blood glucose level or HbA_1c_ (ESM Fig. 2). Daily rhythmicity and monthly variation in AVP-ir, VIP-ir and NT-ir neurons in the SCN did not reach significance, but acrophase and amplitude in daily rhythmicity were significant in the AVP-ir neurons in non-diabetic control individuals (ESM Figs. 3, 4). The overall numbers of AVP-ir and VIP, but not NT-ir, neurons, were significantly reduced in the SCN of type 2 diabetic individuals compared with control individuals (Fig. 1). Furthermore, compared with that in the control individuals, the relative intensity of AVP-ir was significantly lower in type 2 diabetic individuals (Fig. 1d), indicating a decrease in cellular AVP protein expression in type 2 diabetic individuals. This decrease was not observed for VIP-ir and NT-ir neurons (Fig. 1 h,l). High blood pressure was diagnosed in a large number of control donors and type 2 diabetic donors, especially those receiving insulin treatment (ESM Table 1). Previous studies have found decreased numbers of AVP-ir, VIP-ir and NT-ir neurons in individuals with hypertension [9]; however, we only found significant reductions in AVP-ir and VIP-ir neurons, but not NT-ir neurons, in individuals (non-diabetic and type 2 diabetic individuals combined) diagnosed with high blood pressure (ESM Fig. 5), indicating the reductions in AVP-ir and VIP-ir neurons might be related to insulin resistance rather than high blood pressure. Previous studies also showed a reduction in *AVP* mRNA expression in the SCN of individuals that had received corticosterone treatment [10]. In our study, very few individuals (one non-diabetic donor, four with type 2 diabetes) had received corticosterone treatment. We found no differences in the numbers of AVP-ir, VIP-ir and NT-ir neurons in corticosterone-treated vs non-treated individuals (ESM Fig. 6).

**Fig. 2:**
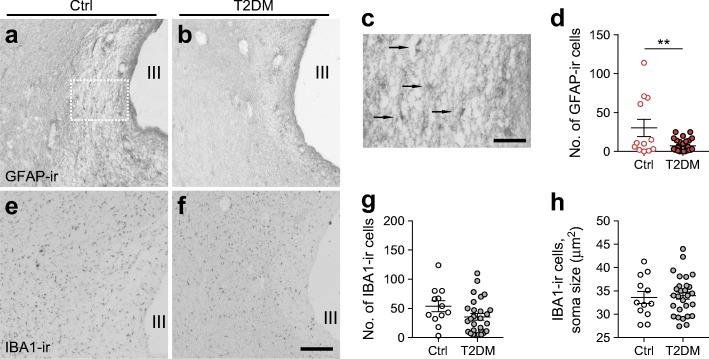
GFAP-ir astroglial cells and IBA1-ir microglial cells in the SCN of non-diabetic and type 2 diabetic individuals. Representative images of GFAP-ir (**a**, **b**) and IBA1-ir (**e**, **f**) in the SCN of non-diabetic control (Ctrl) and type 2 diabetic (T2DM) individuals. (**c**) Higher magnification image of the area framed in (**a**) (arrows point to GFAP-ir cells). Comparison of number of soma of GFAP-ir cells (**d**) and number of soma (**g**) and soma size (**h**) of the IBA1-ir cells in the SCN of Ctrl and type 2 diabetic individuals. Data are presented as mean ± SEM. **p<0.01. III, third cerebral ventricle. Scale bar, 300 μm in (**a**, **b**, **e**, **f**); 100 μm in (**c**)

Overall, the number of cells showing GFAP immunoreactivity was relatively low (Fig. 2a–d) compared with the number of peptidergic SCN neurons. In some individuals, more commonly, type 2 diabetic individuals, very few GFAP-ir cells were detected in the SCN. GFAP-ir cells were only analysed by cell number. For IBA1-ir microglial cells, the number of soma and the soma size (>20 μm^2^) were quantified (Fig. 2e–h). When considering all subjects together, no correlation was found between the number of GFAP-ir astroglial cells and IBA1-ir microglial cells in the SCN and age, post-mortem delay, blood glucose level or HbA_1c_ (ESM Fig. 2d,e). Daily rhythmicity in the number of GFAP-ir astroglial and IBA1-ir microglial cells did not reach significance (ESM Fig. 3). Interestingly, in control individuals, the amplitude of monthly fluctuation of IBA1-ir microglial cell number was more strongly statistically significant than in type 2 diabetic individuals (ESM Fig. 4). Similar to the observations for AVP-ir neurons, the number of GFAP-ir cells in the SCN was reduced in type 2 diabetic individuals (Fig. 2d). However, no correlation was found between the number of AVP-ir and GFAP-ir cells (data not shown). The total number of IBA1-ir microglial cells and their average soma size did not differ between control and type 2 diabetic individuals (Fig. 2g,h). Moreover, no difference was found in GFAP-ir astroglial and IBA1-ir microglial cells between individuals with and without high blood pressure (ESM Fig. 5). Although both astroglial and microglial cells are involved in neuroinflammation [11] and therefore could be affected by corticosterone treatment, no difference was found between corticosterone-treated and non-treated individuals in terms of numbers of GFAP-ir astroglial and IBA1-ir microglial cells (ESM Fig. 6).

## Discussion

In the current study, we performed an analysis of SCN AVP-ir, VIP-ir and NT-ir neurons and IBA1-ir and GFAP-ir glial cells in post-mortem human brain tissue obtained from non-diabetic and type 2 diabetic individuals. Our analysis revealed that the numbers of AVP-ir neurons, VIP-ir neurons and GFAP-ir astroglial cells is significantly decreased in the SCN of type 2 diabetic individuals.

Some major obstacles currently hamper translational studies on brain dysfunction in type 2 diabetic individuals at the molecular level. First, there is no perfect animal model that fully mimics the pathogenesis of type 2 diabetes in humans. Second, although non-invasive brain imaging techniques have provided data on overall changes in brain metabolism in type 2 diabetes, it is poorly understood what these changes mean for specific brain regions and individual cells. In the current study, the unique collection of the Netherlands Brain Bank, with fully informative medical records, gave us the opportunity to retrogradely analyse the medical characteristics of type 2 diabetic individuals and control subjects, and systemically study differences in their brains at the molecular level.

One of the major targets of the SCN projections is the hypothalamic pre-autonomic neurons [12, 13]. The loss of AVP-ir and VIP-ir SCN neurons, therefore, could result in a disbalanced autonomic hypothalamic output, as often observed in type 2 diabetes [14]. Intriguingly, the number of GFAP-ir astroglial cells was reduced in the SCN of type 2 diabetic donors, suggesting that astroglial cells play an important role in maintaining SCN function. Indeed, a recent study demonstrated that in the absence of other cellular clocks, the cell-autonomous astroglial intracellular transcription–translation negative feedback loops alone could drive molecular oscillations in the SCN and circadian behaviour in mice [15].

Previous studies have shown that individuals with type 2 diabetes have a more irregular sleep/wake cycle than the general population [16]. The ‘cause–effect’ question is whether the reduced number of AVP-ir and VIP-ir neurons and GFAP-ir astroglial cells is responsible for the disturbed sleep/wake rhythms or whether the disturbed sleep/wake rhythms affected the SCN. Observations in elderly people and ageing rats suggest the former, since increasing daytime light exposure not only improved sleep/wake rhythms but also increased AVP-ir in the SCN [17]. Nevertheless, whether modifying light exposure can improve the sleep/wake rhythm of individuals with type 2 diabetes and eventually add benefits to their treatment remains to be explored.

In summary, to start to understand the association between circadian clockwork perturbations and the metabolic syndrome in humans, we took advantage of the unique collection of type 2 diabetes human brain tissue in the Netherlands Brain Bank, and systematically analysed SCN cells. Our data indicate that besides regular glucose-lowering medication, normalising circadian rhythms by pharmacological or behavioural approaches might be helpful to treat type 2 diabetes more effectively.

## Electronic supplementary material

ESM (PDF 4330 kb)

## Data Availability

All data generated or analysed during this study are included in this published article (and the accompanying ESM).

## Abbreviations

AVP: Arginine vasopressin
GFAP: Glial fibrillary acidic protein
IBA1: Ionised calcium-binding adapter molecule 1
NT: Neurotensin
SCN: Suprachiasmatic nucleus
VIP: Vasoactive intestinal polypeptide
