# Supplementary material for: Loss of arginine vasopressin- and vasoactive intestinal polypeptide-containing neurons and glial cells in the suprachiasmatic nucleus of individuals with type 2 diabetes
Source: Diabetologia. 2019 Jul 20;62(11):2088–93. doi: 10.1007/s00125-019-4953-7 (PMC6805798; doi:10.1007/s00125-019-4953-7)
Supplement: Supplementary file 1 — (PDF 4330 kb) [file 125_2019_4953_MOESM1_ESM.pdf]

Electronic supplementary material (ESM)

ESM methods

Immunohistochemistry

Hypothalamic tissues were emulsion fixed in 10% phosphate-buffered formalin. Fixation time between groups was similar (Ctrl: 50.00±4.2 hours, T2DM: 48.57±2.7 hours, p=0.773). Tissues were paraffin embedded and coronal sectioned to be 6μm. Every 100th section was used for Nissl staining to pre-determine the anatomical orientation of SCN, that was further analysed by the range of AVP-ir and VIP-ir neurons. For immunohistochemistry, two sections proximate to every 100th section of the estimated SCN boundaries were mounted in an aquadest solution on SuperFrost Plus slides and dried at 37 ºC. Sections were first deparaffinised and hydrated, after which they were rinsed in TBS, and were antigen retrieved for 10 min using microwave treatment at 700W in 0.1 M sodium citrate (pH=6.0), sections were then incubated with: 1) rabbit polyclonal anti-AVP (1:1000) or rabbit polyclonal anti-VIP (1:1000) (produced and validated by Netherlands Institute for Brain Research [1], Amsterdam, the Netherlands) 2) rabbit polyclonal anti-neurotensin (LS-C400910, 1:5000, validated by manufacturer, LifeSpan, LSBio), 3) rabbit polyclonal anti-iba1 (234003, 1:400, Synaptic Systems, Goettingen, Germany)[2], 4) rabbit polyclonal anti-GFAP (N1506, 1:1000, DAKO, USA) [3], in a TBS solution containing 0.25% gelatin and 0.5% TritonX 100 overnight at 4 ºC. In addition, the specificities of these primary antibodies were confirmed by comparison with negative control staining. After rinsing in TBS; sections were incubated with biotinylated horse anti-rabbit IgG antibody (1:400, Vector Laboratories) for 60 min, after rinsing, sections were incubated with avidin–biotin complex (1:800, Vectastain Elite ABC kit; Vector Laboratories Inc.) for 60 min; after rinsing again in TBS, sections were incubated in 0.5 mg/ml 3,3'-diaminobenzidine (Sigma Chemical Co., St. Louis, MO) in TBS containing 0.2% ammonium nickel sulfate (DAB/Ni) (BDH; Brunschwig, Amsterdam, The Netherlands) and 0.01%H2O2 (Merck, Darmstadt, Germany) for approximately 15 min. The reaction was stopped in distilled water. The sections were dehydrated in ethanol and delipidized in 100% xylene. Finally, the sections were cover-slipped using Entellan (Merck, Darmstadt, Germany).

Image analysis

Images of all the stained sections were obtained with an EXi Aqua Bio-Imaging Microscopy Camera (EXi Aqua™) (Zeiss Axiovert 200M microscope). Tiled images from the SCN were taken from each subject. These images were analysed in Fiji, an ImageJ distribution (Madison, Wisconsin, USA). The soma number and the intensity of staining of AVP-ir, VIP-ir and NT-ir neurons and iba1-ir and GFAP-ir glial cells were analysed with their immunoreactivity signals. The DAB/Ni precipitated area was masked and soma size was measured by Fiji. The minimal soma size included in the data analysis was set at 80μm^2 for neurons (based on the observation that with such a size a nucleus and nucleolus were still visible) and 20μm^2 for microglia (based on the observation that with such a size a nucleus

was visible). The GFAP-ir astroglial cell number was manually counted due to the dark background of this staining. The soma number and relative intensity of immunoreactivity for AVP-ir, VIP-ir and NT-ir neurons; the number of GFAP-ir astroglial cells and the soma number/soma size for iba1-ir microglia (per section) were quantified by blind analysis.

Statistics

The *Time of death* and *Month of death* were similar between groups (Mardia-Watson-Wheeler test). A possible daily rhythmicity (plots with *Time of death*), and monthly variation (plots with *Month of death*) for the number of AVP-ir, VIP-ir, NT-ir, GFAP-ir and iba1-ir cells in the SCN was assessed using cosinor analysis with SigmaPlot 14.0 software (SPSS Inc, Chicago, IL, USA). Data were fitted to the following regression: $y = A + B\cdot\cos(2\pi(x-C)/24)$ for daily rhythmicity, and $y = A + B\cdot\cos(2\pi(x-C)/12)$ for monthly variation, where A is the mean level, B the amplitude and C the acrophase of the fitted rhythm. An overall p value (main p-value, P_m) below 0.05 was considered to indicate a significant 24h-rhythmicity or monthly variation [4].

References

[1] Van der Woude PF, Goudsmit E, Wierda M, et al. (1995) No vasopressin cell loss in the human hypothalamus in aging and Alzheimer's disease. Neurobiol Aging 16(1): 11-18.
[2] Chen X, Kelemen SE, Autieri MV (2004) AIF-1 expression modulates proliferation of human vascular smooth muscle cells by autocrine expression of G-CSF. Arterioscler Thromb Vasc Biol 24(7): 1217-1222.
[3] Eggers SD, Horn AK, Roeber S, et al. (2015) Saccadic Palsy following Cardiac Surgery: Possible Role of Perineuronal Nets. PLoS One 10(7): e0132075.
[4] de Goede P, Sen S, Su Y, et al. (2018) An Ultradian Feeding Schedule in Rats Affects Metabolic Gene Expression in Liver, Brown Adipose Tissue and Skeletal Muscle with Only Mild Effects on Circadian Clocks. Int J Mol Sci 19(10).

ESM Table 1 Clinico-pathological data of patients and matched controls.

	Sex	PMD	TOD	MOD	Age	Glucose	HbA1C	Braak stage	HBP	Insulin treatment	Cause of death and clinical diagnosis
**Control**											
1997-065	f	14.50	18:00	5	76	/	/	/	yes	no	Myocardial infarction, unilateral nephrectomy and adrenalectomy.
2000-072	m	18.00	17:00	6	78	1.4	/	1	yes	no	Kidney failure, dehydration, heart failure, renal insufficiency.
2001-021	m	7.67	02:50	2	82	/	/	1	yes	no	Heart attack, ischemic heart disease, kyphosis of backbone.
2007-088	f	5.20	05:00	12	82	4.3	/	3	yes	no	Cachexia, cardiac failure, encephalopathy, mitral valve insufficiency.
2009-022	f	2.92	18:50	3	77	/	/	1	yes	no	Pulmonary metastasis of vulva carcinoma.
2009-039	m	12.92	05:00	5	82	7.4	/	3	yes	no	Heart failure, prostate carcinoma.
2009-095	f	7.17	00:55	12	71	/	31	1	yes	no	Renal failure, CVA, hypertensive retinopathy.
2010-013	m	6.25	17:15	2	70	/	31	0	no	no	Acute myocardial infarction, prostate carcinoma.
2011-082	f	5.92	08:15	10	84	6.2	41	2	yes	no	Respiratory failure, angina pectoris, mitralis valve insufficiency.
2012-005	f	5.60	15:44	1	84	7.5	42	2	yes	no	Heart failure, metastatic breast cancer, scoliosis.
2012-033	f	5.70	22:30	4	95	/	/	3	no	no	Heart failure, cachexia and dehydration, pulmonary disease.
2012-104	m	6.50	10:00	10	79	7.4	/	2	no	no	Legal euthanasia, Ischemic colitis, heart failure with dyspnoea.
**T2DM**											
1989-032	m	4.08	01:30	3	84	/	/	/	no	no	Heart failure, intestinal tumour.
1995-008	f	2.08	20:25	1	79	/	/	3	no	no	Dehydration, endometrium carcinoma.
1995-078	f	6.25	03:50	8	80	/	/	2	yes	no	Dehydration, angina pectoris.
1997-088	f	4.25	15:20	7	78	/	49	1	yes	yes	Pneumonia, seizures, CVA, cortical dysarthria syndrome.
1998-112	f	9.33	06:00	8	84	/	/	3	yes	yes	Pulmonary emboli, CVA, atherosclerosis.
1998-126	m	6.00	10:00	8	71	6.3	/	2	no	yes	Respiratory insufficiency, lung carcinoma.
1999-015	f	2.58	04:05	2	93	/	/	3	no	no	Pneumonia, dehydration, breast cancer.
2001-061	f	4.33	15:00	5	85	5.4	/	2	yes	yes	Myocardial infarction, parkinsonism and depression.
2003-054	m	4.50	20:30	6	67	/	126	1	yes	yes	Cardiac shock CVA.
2004-085	f	4.58	08:05	12	71	5.8	/	3	no	no	Dehydration, CVA with right side paresis.
2005-027	f	4.33	23:40	4	64	8.8	40	0	yes	yes	Respiratory failure, CVA.
2006-033	m	5.00	00:20	4	79	6.6	/	1	no	no	Pneumonia, dehydration, CVA, choledochus carcinoma.
2007-061	f	5.33	12:50	9	83	5.1	/	3	yes	yes	Cachexia, CVA, arteriosclerosis.
2008-061	f	5.00	07:00	7	62	8	79	1	no	no	Cachexia, hyperthyroidism.
2008-105	f	3.87	00:10	12	89	6.6	44	3	yes	no	Pneumonia, coronary artery bypass, atrial fibrillation.
2009-091	m	7.33	13:55	11	84	9.6	62	1	yes	yes	Anaemia, colon and prostate carcinoma metastasis.
2009-096	m	8.42	01:45	12	92	6.7	/	4	no	no	Heart failure, myocardial infarction.
2009-104	m	6.40	16:15	12	87	3.1	/	1	yes	yes	Kidney carcinoma, CVA.
2010-046	f	6.50	15:50	5	88	/	/	3	yes	yes	Dehydration, CVA, ischemic attack.

2010-092	m	5.08	05:45	9	86	/	/	3	yes	no	Dehydration and cachexia, CVA.
2011-027	m	3.30	18:32	3	80	5.5	44	1	yes	yes	Pneumonia, CVA, ischemic attack.
2012-049	f	7.58	04:45	5	70	/	/	2	yes	yes	Cachexia, pancreas carcinoma.
2012-088	f	6.42	20:35	8	85	9.2	/	3	yes	yes	Legal euthanasia, hypoparathyroidism.
2012-092	m	5.75	10:50	8	90	/	/	2	no	no	Prostate carcinoma, CVA.
2012-118	m	4.17	15:30	11	96	/	45	4	no	no	Transient ischemic attack, urinary tract infection, diabetic retinopathy.
2014-040	m	5.17	10:10	7	85	/	82	3	no	no	Pulmonary carcinoma, pneumonia, Arteritis temporalis.
2014-051	m	7.75	11:30	9	92	/	49	3	yes	no	Liver cirrhosis ascites and anuria, hepatic cirrhosis.
2014-063	f	7.58	15:15	10	93	/	83	2	yes	no	Heart failure, renal insufficiency.
**P value**		0.008	ns	ns	0.490	0.727	0.063	0.154			

PMD: post-mortem delay between time of death and time of autopsy (hours), TOD: time of death, MOD: Month of death, HBP: high blood pressure, CVA: cerebrovascular accident. ns: not significant (by Mardia-Watson-Wheeler test). P value was obtained by Tukey's multiple comparison test following one-way ANOVA analysis.

ESM figures and figure legends

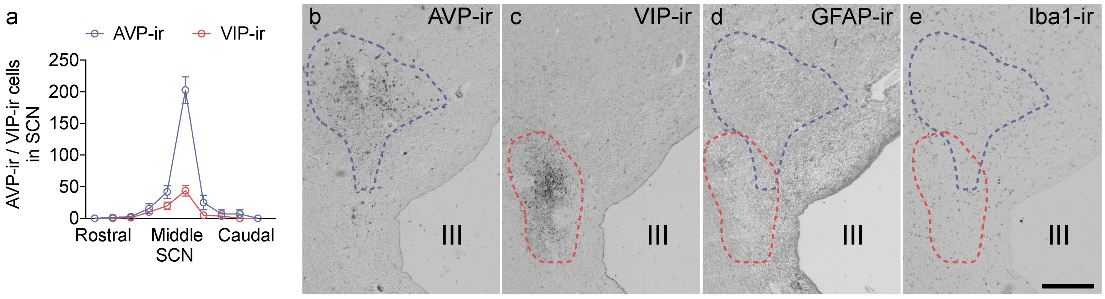

ESM Figure 1. The quantification strategy. (a) The distribution of arginine vasopressin immunoreactive (AVP-ir) and vasoactive intestinal polypeptide immunoreactive (VIP-ir) neurons along the rostral to caudal axis of the suprachiasmatic nucleus (SCN) of control subjects. (b) The AVP-ir area in the dorsal part of the SCN is framed by a blue line. (c) The VIP-ir area in the ventral and central parts of the SCN on the consecutive section is framed by a red line. The glial fibrillary acidic protein immunoreactive (GFAP-ir) astroglial cells (d) and the ionized calcium-binding adapter molecule 1 immunoreactive (iba1-ir) microglia (e) are analysed in the merged area framed by the blue and red lines. Data are presented as mean ± SEM. III: third cerebral ventricle. Scale bar: 500µm.

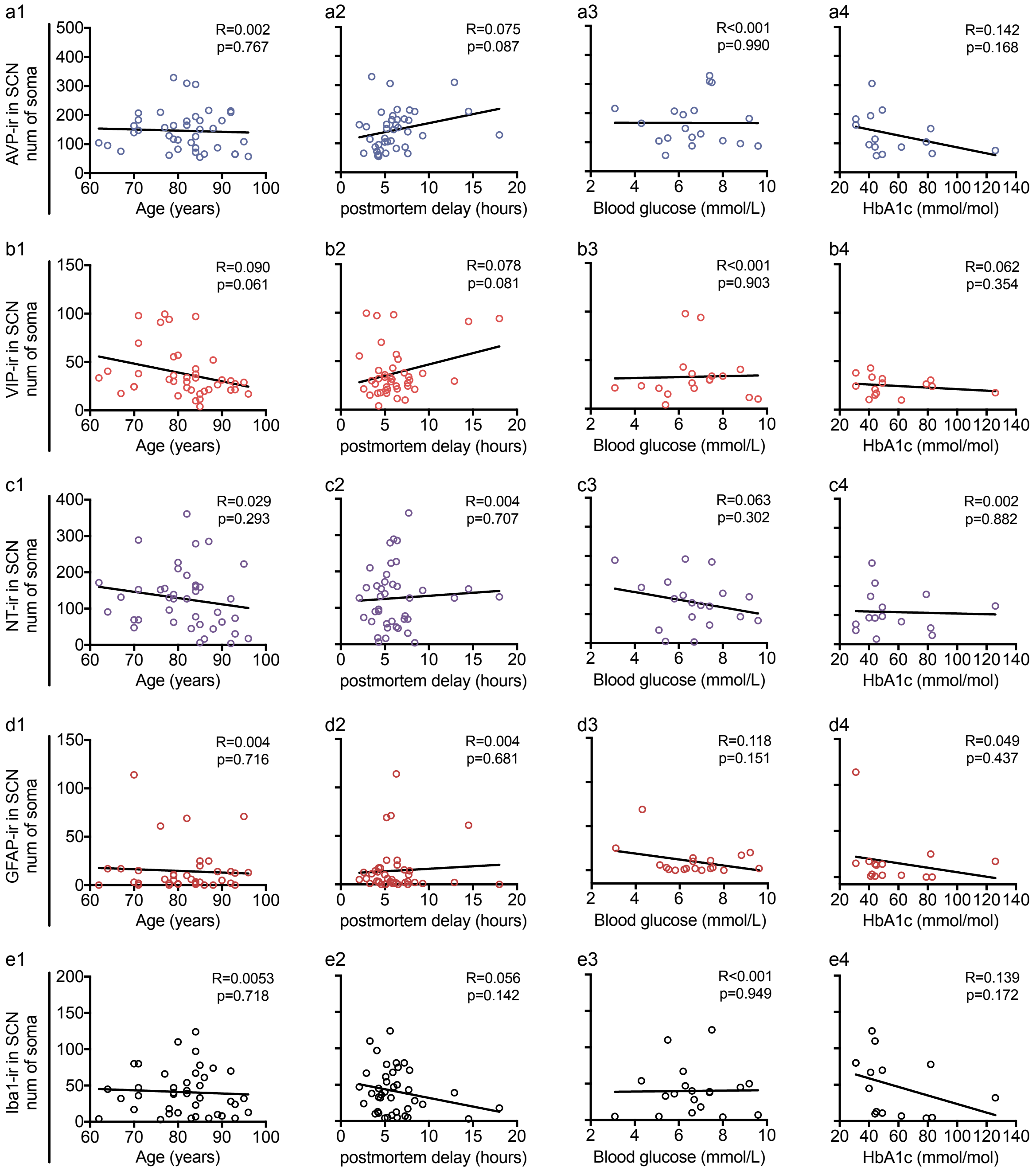

ESM Figure 2. Plots of the number of arginine vasopressin immunoreactive (AVP-ir), vasoactive intestinal polypeptide immunoreactive (VIP-ir), neurotensin immunoreactive (NT-ir), glial fibrillary acidic protein immunoreactive (GFAP-ir) and ionized calcium-binding adapter molecule 1 immunoreactive (iba1-ir) cells in the suprachiasmatic nucleus (SCN) according to Age (a1, b1, c1, d1, e1), post-mortem delay (a2, b2, c2, d2, e2), post-absorptive blood glucose (a3, b3, c3, d3, e3), and HbA1c levels (a1, b1, c1, d1, e1). Data for Age and post-mortem delay concern all subjects, i.e., control and T2DM. Data for post-absorptive blood glucose and HbA1c levels only concern some subjects due to limited availability in both control and T2DM.

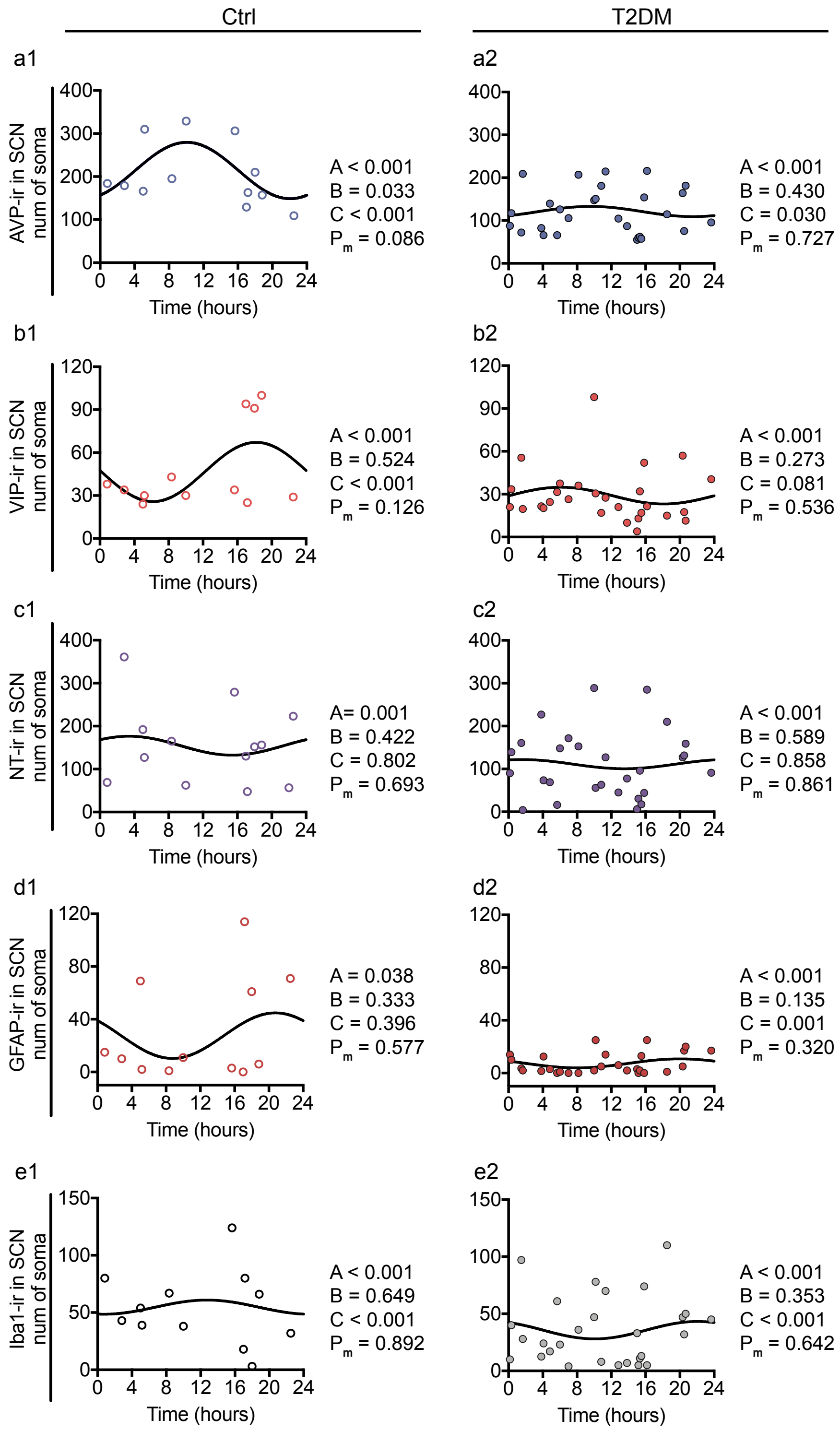

ESM Figure 3. Plots of the number of arginine vasopressin immunoreactive (AVP-ir) (a1-a2), vasoactive intestinal polypeptide immunoreactive (VIP-ir) (b1-b2), neurotensin immunoreactive (NT-ir) (c1-c2), glial fibrillary acidic protein immunoreactive (GFAP-ir) (d1-d2) and ionized calcium-binding adapter molecule 1 immunoreactive (iba1-ir) (e1-e2) cells in the suprachiasmatic nucleus (SCN) according to the *time of death* of the Ctrl and T2DM subjects (A – C: p values of Mean, Amplitude, and Acrophase of cosinor analysis, respectively, P_m: main P value of rhythmicity).

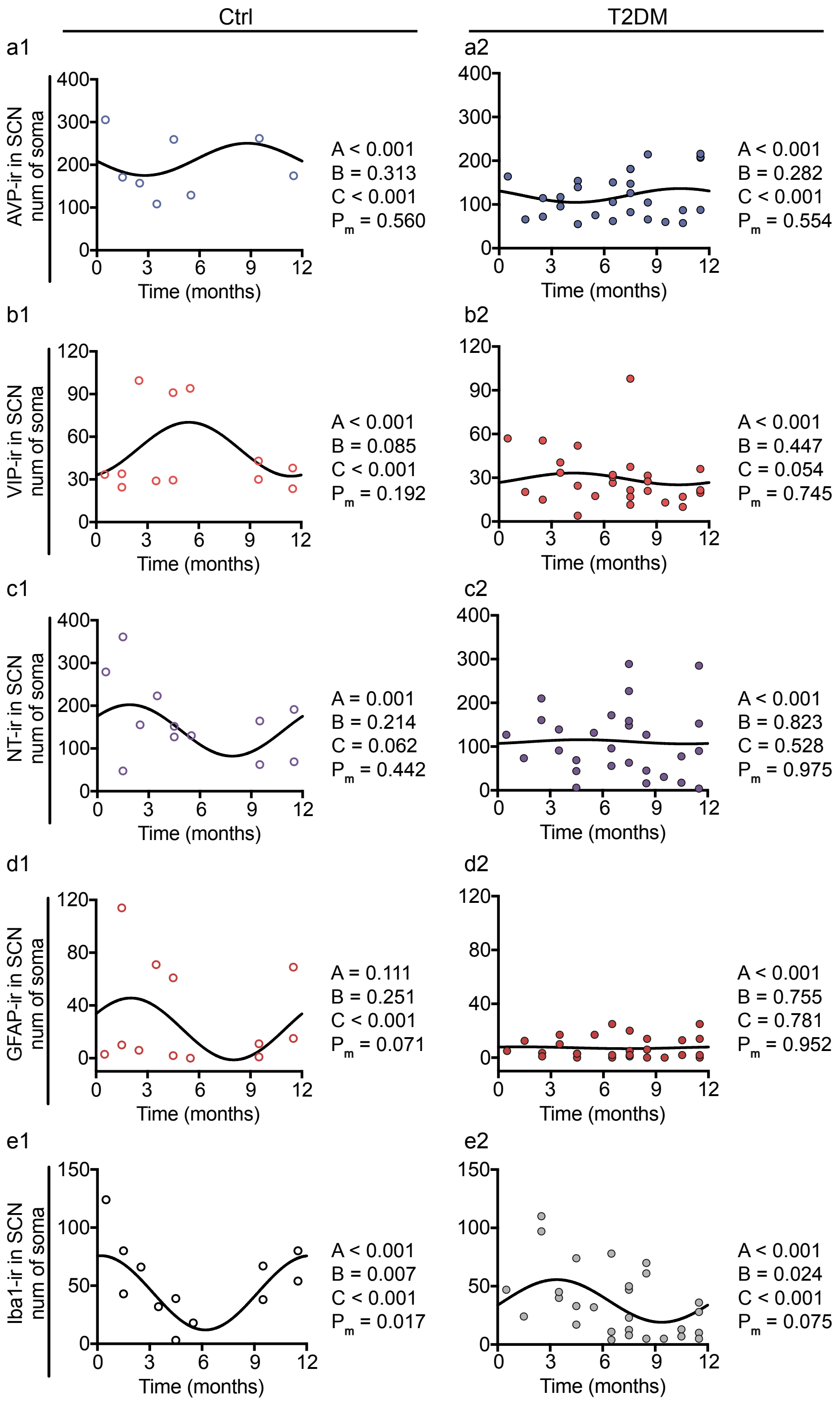

ESM Figure 4. Plots of the number of arginine vasopressin immunoreactive (AVP-ir) (a1-a2), vasoactive intestinal polypeptide immunoreactive (VIP-ir) (b1-b2), neurotensin immunoreactive (NT-ir) (c1-c2), glial fibrillary acidic protein immunoreactive (GFAP-ir) (d1-d2) and ionized calcium-binding adapter molecule 1 immunoreactive (iba1-ir) (e1-e2) cells in the suprachiasmatic nucleus (SCN) according to the *month of death* of the Ctrl and T2DM subjects (A – C: p values of Mean, Amplitude, and Acrophase of cosinor analysis, respectively, P_m: main P value of rhythmicity).

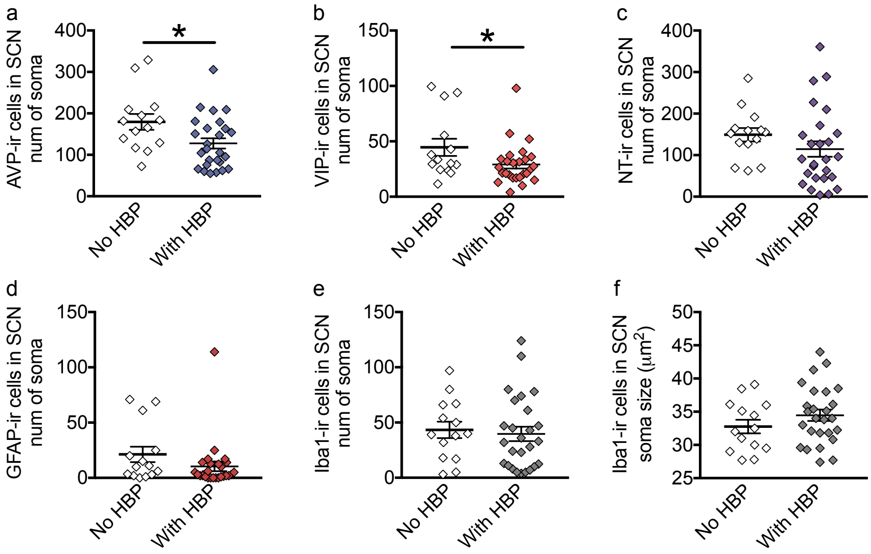

ESM Figure 5. Comparison of arginine vasopressin immunoreactive (AVP-ir) (a), vasoactive intestinal polypeptide immunoreactive (VIP-ir) (b), neurotensin immunoreactive (NT-ir) (c), glial fibrillary acidic protein immunoreactive (GFAP-ir) (d) and ionized calcium-binding adapter molecule 1 immunoreactive (iba1-ir) (e,f) cells in the suprachiasmatic nucleus (SCN) of the subjects that had high blood pressure (HBP) or had no HBP. Data are presented by mean ± SEM. * P<0.05.

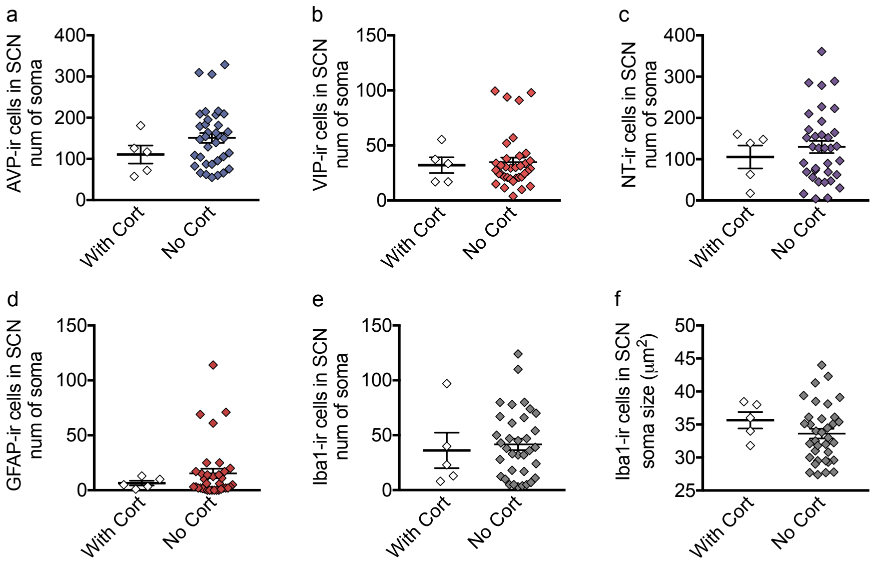

ESM Figure 6. Comparison of arginine vasopressin immunoreactive (AVP-ir) (a), vasoactive intestinal polypeptide immunoreactive (VIP-ir) (b), neurotensin immunoreactive (NT-ir) (c), glial fibrillary acidic protein immunoreactive (GFAP-ir) (d) and ionized calcium-binding adapter molecule 1 immunoreactive (iba1-ir) (e,f) cells in the suprachiasmatic nucleus (SCN) of the subjects that received corticosterone (Cort) treatment or did not receive corticosterone treatment. Data are presented by mean ± SEM.
